# Inversion of the Uterus

**Published:** 1864-02

**Authors:** Morton M. Eaton

**Affiliations:** Peoria, Ill.


### INVERSION OF THE UTERUS.

By MORTON M. EATON, M. D., of Peoria, Ill.

Having never had in my own practice but two cases of perfect inversion of the womb, I would not make any remarks on the subject, had I not been invited so to do. One case I successfully treated about six months since, the other has but recently come under my notice, and I have as yet made no effort to effect its reduction. I believe this accident to be of unfrequent occurrence. I recollect some four years and a half since, being called to a lady hastily; the messenger stating that the womb had come down between her legs, when, on examination, I found it to be a case of miscarriage, with a breech presentation.

And statements of the common people asserting that Mrs. So and So's womb came out of her body turned inside out, and went back itself, etc., are to be credited about as much as when they tell of having their eyes "taken out, scraped and replaced by some noted oculist, with great benefit to the sight."

Most physicians are too timid in attempting to act when unlooked for emergencies arise, and are slow to undertake a case of unusual occurrence.

For *this* reason, patients often loose their lives, and VERY FREQUENTLY put themselves to great expense and trouble to be treated by some physician in a distant city, when in very many cases, if the family physician had possessed a little more energy and enterprise, his patient would have recovered as well and saved considerable money and time, and the physician gained some reputation and experience, and thereby been encouraged to renewed application in the duties of his profession.

Hence I report this case, not to attempt to disprove that it is usually difficult to treat inversion of the uterus successfully, but to encourage the timid, that if they use a little energy, many times their success will far exceed their expectations, and what they had thought difficult would be easily accomplished.

Mrs. S., of W—— st., in this city, called on me at my office, about six months since, stating that she was the mother of three children, the youngest about two months of age; that her confinement had been easy and natural. She was attended by her family physician; nothing unusual occurred; that about a week afterward, she walked about the room, and while doing so a tumor came down between the thighs; that she had tried to press it back, but could not, and she had called to enquire if it should be removed. She could hardly walk from the inconvenience the tumor gave. I examined the tumor and found it to be an inverted uterus, hanging 3½ inches external to the external genitalia, and of the size of my two fists. I informed her of its nature, and proceeded at once to reduce it. I oiled the organ with some Ol. Ricini and my hand as well, and indented the most depending portion with my two front fingers, and finding that I could partially reinvert the womb, I formed my fingers into the form of a cone and making continuous and gentle pressure for several minutes I found I was slowly, but surely reducing the inverted organ, and in about ten minutes had the satisfaction of feeling the fundus of the womb spring from before my fingers with a jerk and of feeling the Os Uteri in situ.

I filled the vagina with sheet cotton and sponge, and applied a perineal bandage, directing her to retain it there till the next day, then to remove it and wash out the vagina with a weak astringent lotion, and re-apply some cotton and the bandage, and repeat this each day. She did so and has had no trouble since. The wash was used about two weeks.

